# Toward mindfulness in quality-of-life research: perspectives on how to avoid rigor becoming rigidity

**DOI:** 10.1007/s11136-016-1492-2

**Published:** 2017-01-09

**Authors:** Mirjam A. G. Sprangers, Carolyn E. Schwartz

**Affiliations:** 1 Department of Medical Psychology, Academic Medical Center, University of Amsterdam, Meibergdreef 15, 1105 AZ Amsterdam, The Netherlands; 2 DeltaQuest Foundation, Inc., Concord, MA USA; 3 Departments of Medicine and Orthopaedic Surgery, Tufts University School of Medicine, Boston, MA USA

**Keywords:** Quality of life, Research principles, Research cycle, Guidelines, Overview

## Abstract

**Background:**

The field of quality-of-life (QOL) research has matured into a discipline with scientific rigor, sophisticated methods, and guidelines. While this maturation is laudable and needed, it can result in a limiting rigidity. We aim to highlight examples of practices that are based on shared research values and principles that, when dogmatically applied, may limit the potential impact of QOL research.

**Methods:**

By juxtaposing rigorous standards with their rigid application for different stages of the research cycle, we suggest more balanced approaches.

**Results:**

Rigidity in cultivating a research question relates to constraining our thinking, leading to ‘safe’ research focusing on small variations of similar studies. Rigidity in operationalizing key constructs focuses on problems with validation practices that hinder further innovations, the use of static questionnaires when a more flexible approach is needed, dismissing rarely endorsed items that are clinically relevant, use of insensitive generic measures when specific measures are required, and a rigid emphasis on short questionnaires. Rigidity in data analysis relates to an undue emphasis on delineating primary and secondary outcomes and an unquestioned insistence on reducing Type 1 errors regardless of the research context. Rigidity in research infrastructure focuses on the unquestioned validity of patient input on scientific matters, and increasingly rigid guidelines and checklists that end up driving grant applications.

**Discussion:**

It is hoped that this overview will lead to a reconsideration of a more flexible application of research principles while retaining scientific rigor.

## Introduction

“The enemy of science is not religion …

The true enemy is the substitution of thought, reflection, and curiosity with dogma.”

Excerpt from The Bonobo and the Atheist by Frans de Waal, 2013 [1].

Over the course of professional training, researchers have been taught rigorous research methods. Some are context specific while others are facilitated and enhanced by guidelines and protocols that are based on shared values, i.e. sets of beliefs about good and bad research, and research principles, i.e., rules about how to conduct good research. The availability of a myriad of such resources is a reflection of the maturation of the field of quality-of-life (QOL) research. While these guidelines and protocols are helpful, we note that they can take on a prominence that can undermine our research. An automatic or ‘unmindful’ application of these value-based research principles can indeed become dogmatic, and may limit the impact of QOL research. At the very soul of scientific inquiry, ‘discovery’ or progress cannot be achieved by blindly following prescriptive scientific methods or just following what has already been established [2, 3]. We aim to highlight examples from different stages of the research cycle and to suggest more balanced approaches. While some dogmatic applications are more pronounced, others are less obvious. We also notice more frequent dogmatic applications in some phases of the research cycle (e.g., operationalization) than in others (e.g., analysis). Our ultimate goal is to encourage more sensible and flexible applications of research principles in practice while retaining scientific rigor.

### Rigor versus rigidity in cultivating a research question

Most research is iterative, with small leaps forward that build on and acknowledge past research and inspiration. As a consequence, reviewers and editors require that we reference precedent papers for the rationales of our studies. When rigidly applied, ‘safe’ research would focus on replications or small variations of the same study (‘me-too studies’). Examples are the description of QOL in yet another specific disease population or validation studies of the same questionnaire in different languages and countries.True replication studies are needed to solidify the field, but they are rare, such as cross-validation studies of the structural validity or measurement invariance of widely used QOL questionnaires. When publications claim to address new concepts they more often than not ‘sell old wine in new bottles’, frequently because their literature search did not extend beyond their own specific niche. For example, measures are published each year that are claimed to be ‘new’, but rarely address new concepts not already assessed by previously published instruments. A more extreme dogmatic application that we have witnessed in research team discussions and as reviewers is that thinking can be constrained to only that which can be referenced by another published work in one’s own niche. It is unfortunate that the resulting iterative studies are generally more easily accepted for publication than unconventional and possibly seminal work, particularly if it challenges widely held beliefs. A more balanced approach would allow for new thinking that may not have a reference to prior work, i.e., paradigm shifts and other innovations, in addition to pursuing more main-stream studies based on ample references. Fortunately, established journals are increasingly creating platforms that explicitly encourage such new thinking.

Similarly, most would agree that any review of the literature must be up-to-date with the latest findings. A drawback of the more rigid application of this principle is that research papers often focus exclusively on recent papers. We would argue that a review of the literature should include relevant papers, including seminal papers of old (do not reinvent the wheel) and hot off-the-press papers (stay current). While this may seem self-evident, we recurrently find ourselves, as a reviewer or editor, alerting authors to (seminal) papers that are missing from the introduction or discussion sections. By balancing more and less recent papers, the authors are acknowledging original work, highlighting possibly neglected ideas that may deserve more attention, providing a historical background, and enhancing our understanding of the field. We would also encourage acknowledging the inspiration by other authors, referencing personal communication and referring to relevant research in other areas of expertise. Such practices will likely advance our field.

### Rigor versus rigor mortis in operationalizing key constructs

A strong research principle is that measures should be validated to ascertain their psychometric integrity and facilitate comparability across studies over time. A related principle is the need to improve the validity of such measures via revisions. Validation studies often reveal minor limitations of the measure. However, the implementation and computing costs are often substantial, reporting and keeping track of the different versions problematic, and the fear of compromising the comparability across studies paralyzing. Thus, tweaking the measure in accordance with the published limitations is rarely feasible. A dogmatic albeit common practice is to adhere to the measure as originally validated, thereby letting the limitations fester quietly in the discussion sections with no improvements made.

Interestingly, validation of a measure is meant to be a multi-dimensional and iterative process until it yields sufficient levels of the desired aspect of validity (e.g., content or criterion validity). Yet, we often see ‘validity’ discussed as if it were a single, dichotomous construct. A measure is valid or not; a measure is never described as valid for 80 or 47%. Moreover, textbooks [4] and widely adopted guidelines [5] ascertain that a single study does not prove a measure to be ‘valid in general’, but for a specific purpose in a specific sample. A specific measure’s few validation papers are referenced to legitimize its further use in applications for which it was not originally validated. Thus, treating validation like a dichotomous construct allows us to say a measure yields ‘good validity’, and creates a belief in a tool irrespective of its application. This practice hinders further innovations. This ‘ritual dance of validation’^1^ needs to be replaced by a more flexible validation practice paying credit to both rigor and adaptability.

For example, the accelerated release of targeted therapies with varying toxicity profiles demands such flexibility. Adaptive clinical trial designs are increasingly being used with the aim to more quickly identify drugs with therapeutic effect. In such designs the protocol is modified (e.g., dosing levels) throughout the trial, based on observations. The sole use of static, standardized questionnaires may fail to ensure content validity in those new clinical trials [6]. These measures need to be supplemented with specific questions relevant to the trial phase. Current discussions in cooperative groups and the United States (US) Food and Drug Administration (FDA) are even leaning toward a modifiable, ‘à la carte’^2^ approach to QOL measurement for efficacy trials. For example, patients might be asked different questions on-treatment than during follow-up [7]. Paying more attention to flexibility and clinical relevance will likely make the validation process less ceremonial and enhance its meaningfulness.

Related to the issue of clinical relevance, we note the dilemma of rarely endorsed items in patient-reported outcome tools. While it is true that measurement validation approaches in classical test theory analyses work better when items are not skewed, dropping rarely endorsed items (i.e., skewed) has a cost. It has been documented that dismissing rarely endorsed items can reduce the ability of a measure to detect important differences between subgroups or identify clinical syndromes. For example, about half of the items in the Missoula-Vitas Quality of Life Index are highly skewed, suggesting rare endorsement. Its factor structure is unstable across illness groups and its internal-consistency reliability scores are low [8]. These poor psychometric indicators coupled with its highly rated relevance from both patients and healthcare providers suggest that the tool serves a useful clinical function but it is not feasible as a psychometric outcome measure [8]. The shared value that we need reliable instruments, particularly when used as primary endpoints or as the basis for decision-making, may turn into a rigid neglect of their clinical relevance. Therefore, rarely endorsed items should be examined and retained if clinically or scientifically important. An acknowledged caveat of keeping those items is that they cannot be retained in a subscale as they would reduce its internal consistency reliability, and thus would be analyzed as single items at the cost of lower reliability.

A sign of success for our field is that there are widely accepted standards for the collection and reporting of QOL by regulatory agencies, e.g., FDA [9], and by the National Institutes of Health (NIH) [10]. Moreover, recommendations for incorporating QOL in prospective comparative effectiveness studies (e.g., clinical trials) have been established [11]. It is generally acknowledged that generic measures lack the sensitivity to detect differences in toxicity profiles and side effects between treatment arms. This insight led to the development of disease-specific questionnaires [4]. Nonetheless, there are still trials that only employ generic measures [12]. This trend does not belong to the past; we currently witness the same practice as committee members reviewing trial protocols. Moreover, whereas European Organisation for Research and Treatment of Cancer (EORTC) clinical trials consistently administer the core Quality of Life Questionnaire (QLQ-C30), they rarely implement site- and treatment-specific modules because their specialized nature is more difficult to fit into trial objectives [7]. A related trend is the predilection toward generic or “off-the-shelf” QOL assessments where a clinical trial team can simply download a standard questionnaire or template with guidelines and insert it in the protocol [7]. Whereas generic measures have some advantages, they may risk missing the difference across disparate therapies [6]. Further, using solely generic measures backfires on our field; it has led to the idea that assessing QOL in clinical trials is not needed since it does not make a difference. Using such generic measures exclusively may be a halfhearted compromise to address QOL. It is also a sign of undue reliance on measures validated in another context at the cost of ensuring content validity. It is akin to the parable of the person who looks for lost keys under the street light rather than where he lost them. It is better to supplement generic measures with content-valid QOL questions. Calibrated item banks (e.g., [13]), may also be useful as they cover the entire spectrum of functioning or symptom experience. However, they may not include all the domains needed for specific trials. Whether existing or new, one should select questions that are most likely to reveal clinically important differences between treatment arms based, at a minimum, on their toxicity profiles.

While content-valid measurement is a long-held goal, it is often counteracted by a concern about excessive patient burden as, in general, the longer the questionnaire, the lower the response rate [14]. Our experience is that grant reviewers, ethics committees and clinicians alike resist study protocols with numerous questionnaires. They state that the perceived patient burden and the resulting risk of low-quality data outweigh the need for measuring theoretically or clinically relevant constructs. This focus on short questionnaires might be a sign of a latent mistrust of questionnaire data: “OK, we do not really think that this will show anything of importance, so please make it quick.” This attitude is in marked contrast to entire fields of research (e.g., psychology) or even FDA guidelines that require high-quality assessments of subjective experiences. A common research practice is to limit the questionnaire to a 10–15 min completion time for clinical trials [11] to ensure participant compliance and few missing data. Such a general rule of thumb does not exist for standalone studies. Clearly, computer adaptive testing or IRT-based short forms allow for administering only relevant and fewer items without losing scientific rigor. Nonetheless, many studies require longer questionnaires. The experience of many researchers is that long questionnaires can be successfully implemented if the respondents are interested and engaged in the topics [15–18]. In fact, patients often welcome QOL questionnaires as a sign of interest in life aspects that matter to them [19]. Moreover, longer questionnaires are more feasible if data collection approaches are used that allow participants to ‘save and continue’ [20]. In the US, study participants are generally more willing to spend time completing long questionnaires if they are paid for their time [20], although cancer patients were found to be unwilling to complete long questionnaires even when paid [21]. Special attention should be devoted to explaining possible redundancy in a questionnaire that consists of multiple instruments, e.g., when similar questions are asked by different instruments [20]. A careful explanation why this redundancy is necessary will likely increase patients’ motivation [20].

### Mindfulness in data analysis

Two other research principles are that statistical power considerations require the distinction between primary and secondary outcomes in testing a priori hypotheses and that statistical testing should limit false rejection of the null hypothesis (Type 1 error). The corollary is the requirement to adjust for multiple comparisons [22] and false discovery rates [23]. Unfortunately, insufficient emphasis on Type 1 error is a recurrent research practice. For example, when an effectiveness study of a psychosocial or medical intervention results in an insignificant primary outcome, researchers too frequently seek and publish significant results from outcomes and subgroups that were not pre-specified, thereby ignoring the risk of false-positive results. This insipid research practice is often done with the excuse that the analyses are “exploratory”. However, publications of randomized clinical trial results are expected to pay credit to Type 1 errors, with prior publication of trial protocols being increasingly required and the establishment of clinical trial units with biostatisticians on board. Conversely, in the context of genuine, exploratory or descriptive research, a rigid emphasis on reducing Type I error may be self-limiting [24]. In those cases, we would argue that one should allow for the possibility that a research study has value by describing findings across a range of outcomes and reporting the effect size estimates. By the same token, publications of non-significant (replication) findings are needed to build an evidence-based body of research [25].

### Fashion versus fusion in shared perspectives

The long-held core value that QOL is inherently subjective gains prominence in an increasingly patient-centered health care environment. Our measures amplify the patient perspective. If a patient and a proxy diverge in assessing the patient’s QOL, it is almost a moral imperative that the patient is ‘right’. For example, discrepancies between patient-proxy ratings are interpreted, a priori, as evidence of the inaccuracy or biased nature of proxy-generated data, even if the patient is intellectually impaired [26]. However, the level of patient-proxy agreement is also influenced by methodological and psychometric factors. Moreover, patient ratings, such as proxy ratings, may be subject to biases [27]. While a large body of research has shown that both patients and proxies can provide relevant and complementary information about QOL [28, 29], it is less fashionable to consider other uses of proxy assessment. Especially for the physical QOL domain, an exclusive reliance on patient reports might signify procedural rigidity instead of conceptual rigor. For example, the doctors’ perspective on the patient’s symptom experience can serve as a useful benchmark for an individual’s symptom experience relative to others with a similar condition, given their witnessing the entire spectrum of patients. Further, patient-proxy congruence can be used as an indicator of intimacy or social connectedness [30] and thus can be a helpful outcome for behavioral intervention studies [31].

### Consciousness in maintaining the research infrastructure

Thus far we have addressed the research cycle, but before research can even begin one must seek funding. In response to calls for increasing transparency, grant funding agencies adopt grant proposal forms and evaluation criteria that are as specific and uniform as possible. This laudable egalitarian approach has, however, led to the increasing use of rigid time-consuming guidelines and checklists that end up driving grant applications (i.e., the forms seem to take precedence over the science). The shared values of objectivity and comparability seem to have turned into bureaucratic rigidity. Academic research is increasingly dependent on external funding and the probability of getting that funding is often less than 10%. Hence, researchers must devote an arduous amount of time to grant writing despite a very low likelihood of funding. This is a waste of precious resources that most researchers acknowledge but feel powerless to change.

We would urge a fundamental change in the nature of grant proposals. Particularly, in Europe, sponsors increasingly require a relatively brief concept statement (one to three pages) as an initial submission. Similar to the simpler approach taken by pharmaceutical companies in funding academic research, we would suggest that if and only if this concept is compelling and likely to be funded, the researchers should continue. The further fleshing out of the proposal as well as supporting documentation would then be done in an iterative but brief manner to negotiate approaches (i.e., back-and-forth communication) that integrate the sponsor’s mission. The resulting funding agreement would allow the researcher ‘wiggle room’ to figure out the best study procedures while keeping the sponsor apprised of the study’s progress. We believe that embracing the *‘*speed of trust’ [32] is not only more considerate but will result in a better use of resources.

Review committees are also key to funding. It is increasingly acknowledged that patients have a unique understanding of their disease, which should not only be integrated at each stage of the research cycle, but also at the stage of reviewing grant proposals. Their input can be particularly cogent in assessing the study’s relevance and real-life impact the research outcome will have. The above-mentioned moral imperative can also lead to the unquestioned validity of patient input on scientific matters when included as reviewers for funding bodies. While we do not dispute the importance of real-life experience informing patients’ desired subjective input, it should be carefully considered with the same caveats as that of any other stakeholder.

### Going forward

Awareness can be a first step toward change. We would like to emphasize that guidelines and checklists are helpful heuristics, not irrefutable prescriptions. Shared values and research principles may turn rigor into rigidity when they are applied unmindfully. We hope that this brief discussion of current trends will stimulate discussion and serve as a reminder to facilitate a more flexible application of research principles in practice while keeping scientific rigor.

## Footnotes

1 The term ‘ritual dance of validation’ is not ours. MAGS heard it once during a lecture that she thinks Peter Fayers held. When asked, Peter could not remember having used this term. If someone recognizes it as his/hers, please accept our sincere apologies for not referencing appropriately and let us know who you are.

2 The term ‘à la carte’ approach to QOL measurement was coined by Corneel Coens.
